# Niche-driven divergence of prokaryotic and viral communities in *Pogonatum cirratum*

**DOI:** 10.1016/j.isci.2026.117172

**Published:** 2026-08-13

**Authors:** Chao-Jian Hu, Muhammad Asif, Jin-Hui Liu, Cheng-Guang Xing, Xiao-Qing Luo, Wen-Hui Lian, Mei-Xiang Li, Yong Huo, Shu Cao, Jian-Yuan Chen, Wen-Jun Li, Wei-Qiu Liu

**Affiliations:** 1 School of Life Sciences, School of Ecology, Guangdong Provincial Key Laboratory of Plant Stress Biology and State Key Laboratory of Biocontrol, Sun Yat-Sen University, Guangzhou, China; 2 State Key Laboratory of Desert and Oasis Ecology, Key Laboratory of Ecological Safety and Sustainable Development in Arid Lands, Xinjiang Institute of Ecology and Geography, Chinese Academy of Sciences, Urumqi, China

**Keywords:** *Pogonatum cirratum*, prokaryotic, virus, ecological niches, moss-microbe interactions

## Abstract

Ecological niche partitioning shapes microbial communities in terrestrial mosses, yet its underlying mechanisms and associated viral diversity remain poorly understood. Here, we characterized prokaryotic and viral communities in the rhizosphere soil (Rs) and endophytic niche (Pc) of *Pogonatum cirratum* using amplicon and metagenomic sequencing. Rs exhibited higher species richness, co-dominated by *Pseudomonadota*, *Acidobacteriota*, and *Actinomycetota*, whereas Pc was dominated by *Pseudomonadota* (81.13%) but showed greater functional diversity. Source tracking revealed that 10.57% of Pc taxa originated from Rs, suggesting host-mediated filtration of beneficial microbes. Deterministic processes predominantly governed prokaryotic assembly, with iron cycling accounting for ∼14% of total metabolic potential in both niches. Rs contained more biosynthetic gene clusters, while viral communities diverged in taxonomy and auxiliary metabolic genes profiles. These findings demonstrate that *P. cirratum* maintains compartmentalized prokaryotic and viral communities through niche-specific abiotic filtering and biotic selection, promoting nutrient acquisition and stress resilience in bryophyte-dominated ecosystems.

## Highlights

• Amplicon and metagenomics dissect niche-partitioned prokaryotes and viruses in *P. cirratum*
• Viral taxa and auxiliary metabolic genes show clear niche divergence in *P. cirratum*
• BGCs uncover niche-specific microbial chemical defense in moss habitats

## Graphical abstract

**Figure:**
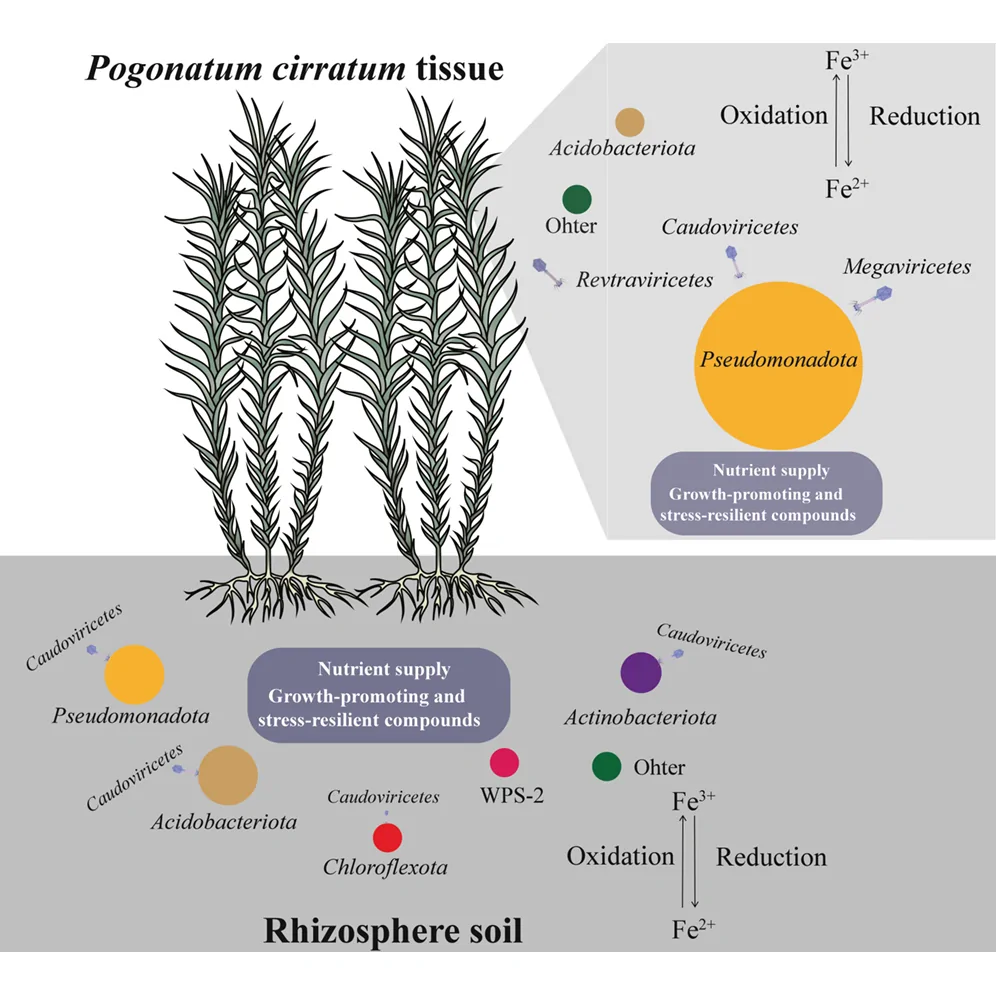

## Teaser

Ecology; Biological sciences; Microbiome; Plant ecology

## Introduction

Mosses represent an ancient lineage of terrestrial plants, with an evolutionary history spanning approximately 500 million years.^1^^,^^2^^,^^3^^,^^4^ Mosses, among Earth’s earliest land plants, thrive in diverse habitats through intricate partnerships with microorganisms. Often referred to as the “amphibians” of the plant kingdom, mosses were among the first plants to colonize terrestrial environments. Despite their primitive origins, they exhibit remarkable species diversity, second only to angiosperms. Mosses are globally distributed and play critical ecological roles as primary producers and pioneers in extreme environments, such as deserts and wetlands.^5^ Their remarkable environmental adaptability and ecological functions are tightly linked to their associated microbial communities, which are instrumental in maintaining moss ecosystem stability.^6^^,^^7^^,^^8^^,^^9^ Their symbiotic relationships, particularly within the rhizosphere and endophytic compartments, are thought to underpin moss resilience and nutrient cycling. Yet, how these distinct ecological niches shape microbial communities in nonvascular plants remains underexplored.

Mosses interact with microbial communities within their tissues and rhizosphere soil, and in turn, these microbes significantly influence the mosses’ growth, development, and ecological functions. Mosses exudates modulate abiotic factors such as soil pH and temperature, creating distinct microenvironments that shape the structure and function of rhizosphere microbial communities.^9^^,^^10^^,^^11^^,^^12^^,^^13^^,^^14^ These microbial communities, in turn, contribute to biogeochemical cycles by regulating soil organic matter, fixing atmospheric nitrogen, and assimilating inorganic phosphorus. Additionally, they produce phytohormones, such as cytokinins and auxins, which promote healthy plant growth and facilitate adaptation to specific habitats.^15^^,^^16^^,^^17^ Endophytic microbial communities, typically recruited from the rhizosphere soil or atmosphere also improve moss growth, stress resistance, and defense against pathogens.^18^^,^^19^^,^^20^^,^^21^^,^^22^ This symbiotic interaction triggers these microbes to produce secondary metabolites that promote the development of moss protonemata,^23^ and inhibit pathogens and ultimately induce resistance mechanisms.^11^^,^^24^

Among bryophytes, *Pogonatum cirratum* stands out as a common and representative moss species in South China. It thrives on roadside forest slopes and exhibits strong environmental adaptability.^25^^,^^26^^,^^27^^,^^28^^,^^29^^,^^30^ However, the diversity, composition, and ecological functions of microbial communities across their various niches remain insufficiently explored. As critical yet frequently overlooked components of ecosystems, viruses exhibit high abundance and diversity across different environments. Notably, they play key roles in organismal evolution and biogeochemical cycles, including the modulation of microbial functions through auxiliary metabolic genes (AMGs).^31^^,^^32^^,^^33^^,^^34^^,^^35^^,^^36^ However, the interactions between plants and their microbial systems are highly complex, primarily because the bacteria associated with hosts comprise a mixed population, many of which are uncultivated and harbor viral diversity. Therefore, multi-omics techniques enable us to further explore plant-microbe interactions, particularly the role played by viruses therein.

As a United Nations Educational, Scientific and Cultural Organization (UNESCO) World Heritage Site, Danxia Mountain Nature Reserve features the unique Danxia landform and harbors a bryophyte flora with quite high species diversity. *Pogonatum cirratum* is one of the dominant moss species in this region, often forms extensive monodominant communities on roadside forest slopes, thus providing an ideal model for investigating niche-driven microbial community divergence across different ecological niches of mosses. To address the aforementioned knowledge gap regarding niche partitioning mechanisms in moss-microbe systems, we hypothesized that the distinct environmental conditions in the rhizosphere soil and endophytic niche will drive significant divergence in the taxonomic composition and functional profiles of both prokaryotic and viral communities. To test this hypothesis, we characterized the microbial communities associated with *P. cirratum* tissue samples and rhizosphere soil collected from Danxia Mountain, Guangdong, China. Specifically, we used 16S rRNA gene amplicon sequencing to investigate the composition and diversity of prokaryotic communities in rhizosphere soil (Rs) and endophytic niche (Pc). We then performed metagenomic sequencing to further extend the diversity of secondary metabolic gene clusters, the composition of viral communities, and their auxiliary metabolic functions across these two contrasting niches. These findings provide a foundational framework for future research on the complex interactions among mosses, microorganisms, and viruses.

## Results and discussion

### Distinct patterns of prokaryotic diversity and community assembly across ecological niches in *Pogonatum cirratum*

**Figure 1:**
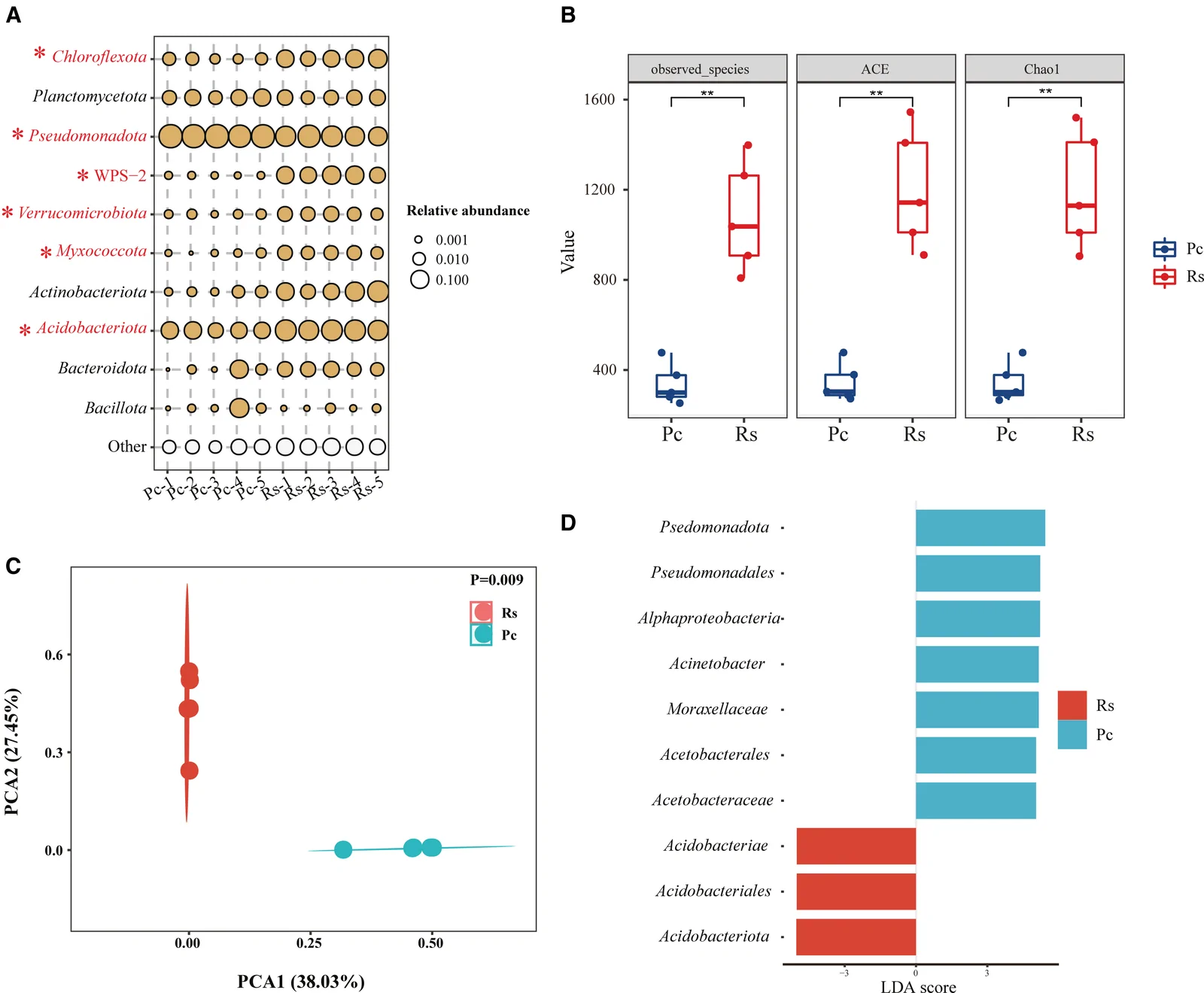
Niche-driven divergence in prokaryotic community structure and assembly Pc: *Pogonatum cirratum* endophytic prokaryotic communities, Rs: rhizosphere soil prokaryotic communities. (A) Mean relative abundance of the top 10 prokaryotic phyla in Rs and Pc. Significant differences in phylum abundance between Rs and Pc, determined by *t* test, are marked with “∗” (*p* < 0.05). (B) Comparison of prokaryotic species richness (observed species, ACE, and Chao1 indices) between Rs and Pc. Statistical significance was tested by Wilcoxon rank-sum test (*p* < 0.01). (C) Principal component analysis (PCA) of prokaryotic communities based on the Bray-Curtis dissimilarity. Wilcoxon rank-sum test confirmed significant structural separation between Rs and Pc (*p* = 0.009). (D) Linear discriminant analysis effect size (LEfSe) identifying differentially abundant microbial taxa between Rs and Pc. Only taxa with a linear discriminant analysis (LDA) score > 5.0 and statistical significance (*p* < 0.05, Wilcoxon rank-sum test) are shown.

Our investigations revealed clear differences in the composition and diversity of prokaryotic communities between rhizosphere soil and the endophytic compartments of *P. cirratum* (Figures 1A–1C). These observations suggest that distinct niche-specific environmental conditions play a vital role in shaping microbial community structures. At the species level, diversity indices (e.g., Shannon and Simpson) showed no notable differences between the two niches, indicating similar levels of diversity and evenness (Figure S1). However, the rhizosphere soil exhibited a higher species richness, as indicated by significantly higher values of observed species, ACE, and Chao1 indices (Figure 1B). This higher species richness suggests that the rhizosphere is a more diverse and dynamic microhabitat. This pattern may be associated with moss-derived exudates (e.g., organic acids, sugars, and amino acids), which support a wide range of microbes adapted to utilizing diverse carbon sources.^10^^,^^37^^,^^38^ Principal component (PC) analysis based on Bray-Curtis dissimilarities highlighted structural distinctions between the rhizosphere soil and endophytic microbial communities (Figure 1C). These two communities formed distinct clusters, with the first two PCs accounting for 65.48% of the total variation. Furthermore, the Wilcoxon rank-sum test confirmed significant differences in community structure between samples from these two habitats (*p* = 0.009).

At the phylum level, the microbial communities in rhizosphere soil were characterized by co-dominance among multiple phyla, with mean relative abundances of *Pseudomonadota* (27.96%), *Acidobacteriota* (27.62%), *Actinomycota* (13.56%), WPS-2 (7.78%), and *Chloroflexota* (7.76%). In contrast, the endophytic prokaryotic community exhibited a markedly skewed composition, dominated by *Pseudomonadota* (81.13%), with *Acidobacteriota* as the second most abundant phylum at a significantly lower proportion (5.67%). Among the top ten dominant phyla, six, namely *Chloroflexota*, *Pseudomonadota*, WPS-2, *Verrucomicrobiota*, *Myxococcota*, and *Acidobacteriota*, exhibited statistically significant differences in relative abundance between the two ecological niches (Figure 1A).

Further analysis using linear discriminant analysis effect size (LEfSe) identified niche-specific enriched microbial taxa: *Pseudomonadota* was significantly enriched in the endophytic compartment, while *Acidobacteriota* was specifically enriched in the rhizosphere soil (Figure 1D). As the dominant phylum in both niches, *Pseudomonadota* was found to promote moss health and defend against pathogens through its functional versatility (e.g., growth promotion, disease resistance).^38^^,^^39^ Predictions from fast expectation maximization microbial source tracking (FEAST) suggested that approximately 10.57% of endophytic microbes were derived from the rhizosphere soil (Figure S2). This indicates that *P. cirratum* selectively filters beneficial microbes from the rhizosphere soil into its endophytic tissues, likely facilitating environmental adaptation and enhancing disease resistance.^16^^,^^40^^,^^41^

The mechanisms governing microbial community assembly further emphasize niche-specific differences. Phylogenetic clustering was observed in both niches, as indicated by a nearest taxon index (NTI) greater than zero (Figure S3A), suggesting that microbial taxa in each niche are phylogenetically more closely related than expected. To quantitatively disentangle the relative contributions of different ecological processes, the standardized null-model-based framework was employed, combining the beta NTI (βNTI) and the Bray-Curtis dissimilarity-based Raup-Crick metric (RCbray). Quantitative analysis confirmed that deterministic processes exerted stronger regulatory control over rhizosphere microbial communities than over endophytic communities (Figure S3B). Specifically, the mean βNTI value of rhizosphere soil microbial communities was −3.35, with 80% of pairwise sample comparisons having βNTI < −2, indicating that rhizosphere community assembly was predominantly driven by homogeneous selection (a deterministic process). The remaining 20% of comparisons with |βNTI| ≤ 2 were attributed to stochastic processes, and further RCbray analysis revealed that all stochastic assembly in the rhizosphere was driven by dispersal limitation (RCbray > 0.95). In contrast, for moss endophytic microbial communities, the mean βNTI value was −2.59, with 60% of pairwise comparisons having βNTI < −2. This demonstrated that homogeneous selection was also the primary assembly process in the endophytic niche, albeit with a substantially weaker selective strength than in the rhizosphere. The remaining 40% of comparisons were governed by stochastic processes: within this stochastic fraction, 25% was driven by homogenizing dispersal (RCbray < −0.95), and 75% was driven by undominated processes (|RCbray| ≤ 0.95).

These findings imply that niche-specific environmental factors select for microbial taxa possessing adaptive traits suited to the local environment, such as efficient nutrient utilization in the nutrient-rich rhizosphere soil and robust host tissue compatibility within the endophytic niche. For instance, Sphagnum mosses maintain a highly specific bacterial community distinct from surrounding peat water, which is enriched in nitrogen fixation and polymer degradation traits to enable efficient nutrient acquisition in oligotrophic habitats.^42^ In parallel, endophytic bacteria isolated from Sphagnum tissues exhibit both plant growth-promoting properties and essential colonization traits, including asymptomatic tissue penetration and phytohormone production that modulates host responses, exemplifying the strong selective pressure for host-compatible microbial traits in the endophytic niche.^43^

### Distinct functional profiles in rhizosphere soil and endophytic prokaryotic communities

**Figure 2:**
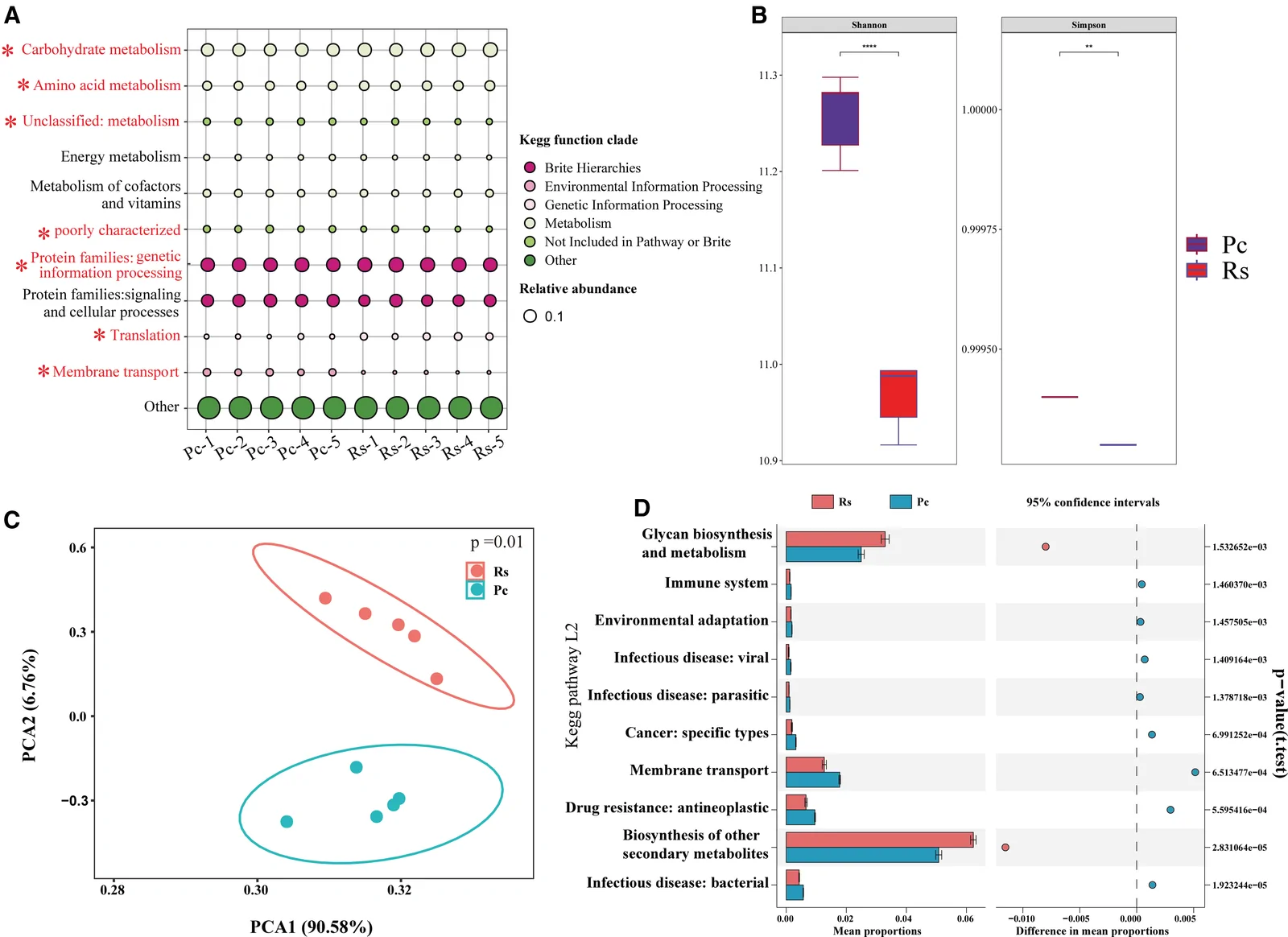
Distinct functional profiles in rhizosphere soil and endophytic prokaryotic communities Pc: *Pogonatum cirratum* endophytic prokaryotic communities, Rs: rhizosphere soil prokaryotic communities. (A) Functional composition of prokaryotic communities at KEGG pathway level 1. Each point represents a functional category, with color-coded modules and size indicating relative abundance. (B) Comparison of functional α-diversity between Rs and Pc. Statistical significance was determined by Wilcoxon rank-sum test (*p* < 0.01). (C) Functional β-diversity analysis via principal component analysis (PCA) based on Bray-Curtis dissimilarities of KO numbers. Ellipses denote 95% confidence intervals, and statistical separation was confirmed by Wilcoxon rank-sum test (*p* = 0.01). (D) Major functional pathways (top 10) with significant differences at KEGG pathway level 2, visualized by STAMP analysis. Bars represent mean proportion of each pathway in Rs (red) and Pc (blue), with dots indicating 95% confidence intervals and *p* < 0.05 by Welch’s *t* test.

Functional predictions using PICRUSt2 revealed distinct metabolic and functional profiles between prokaryotic communities in the rhizosphere soil and endophytic niches of *P. cirratum*. Major functional pathways included brite hierarchies, environmental information processing, genetic information processing, and metabolism, with notable differences in their relative abundances across the two niches (Figure 2A). For instance, genes associated with carbon and nitrogen metabolism (under the metabolism category) were more prevalent in Rs, while genes involved in signal transduction and membrane transport (under environmental information processing) were enriched in Pc. Despite Pc exhibiting lower species richness (Figure 1B), its functional diversity (measured as the alpha diversity of KEGG orthologs) was significantly higher than that of Rs (Figure 2B). This observation was further validated by metagenomic analysis of metagenome-assembled genomes (MAGs) across both niches (Figure S5). Principal component analysis (PCA) based on functional profiles confirmed clear structural separation between Rs and Pc, with the first two axes explaining 90.58% and 6.76% of the variance, respectively (*p* = 0.01, Figure 2C). We hypothesize that endophytic prokaryotes, shaped by stronger host-mediated selective pressures within moss tissues, have evolved a broader functional repertoire to fulfill host-derived demands despite reduced taxonomic diversity.^44^

To further dissect niche-specific functional specialization, we performed functional pathway analysis using STAMP software^45^ at the KEGG Level 2 (KEGG L2) hierarchy, identifying the top 10 pathways with significant differences between Rs and Pc (Figure 2D). In Rs, the biosynthesis of other secondary metabolites and glycan biosynthesis and metabolism pathways were notably enriched (mean proportion higher in Rs, *p* < 0.05). The former supports the production of bioactive compounds (e.g., antibiotics, antifungal agents, and antioxidants),^46^^,^^47^^,^^48^ which likely confer *P. cirratum* with resistance against soil-borne pathogens. The latter facilitates the synthesis and degradation of carbohydrates (e.g., cellulose and pectin), contributing to energy reserves and structural integrity in moss tissues, highlighting the role of prokaryotes in mediating carbon cycling and physical resilience.^49^ Conversely, the endophytic niche showed enrichment in pathways such as “xenobiotics biodegradation and metabolism” and “membrane transport” (mean proportion higher in Pc, *p* < 0.05). These pathways are consistent with endophytes’ adaptations to detoxify xenobiotics within moss tissues and facilitate the exchange of nutrients and ions with the host, respectively. Statistical analysis confirmed the significant niche-specific enrichment of these pathways, a pattern presumably driven by the distinct selective pressures in rhizosphere soil (diverse microbial interactions and nutrient heterogeneity) versus endophytic tissues (host-mediated filtration and constrained nutrient availability). Collectively, these functional divergences are consistent with niche-specific roles of prokaryotes in potentially enhancing *P. cirratum*’s environmental resilience and symbiotic fitness.

### Co-occurrence networks reveal niche-specific patterns in *Pogonatum cirratum* prokaryotic communities

**Figure 3:**
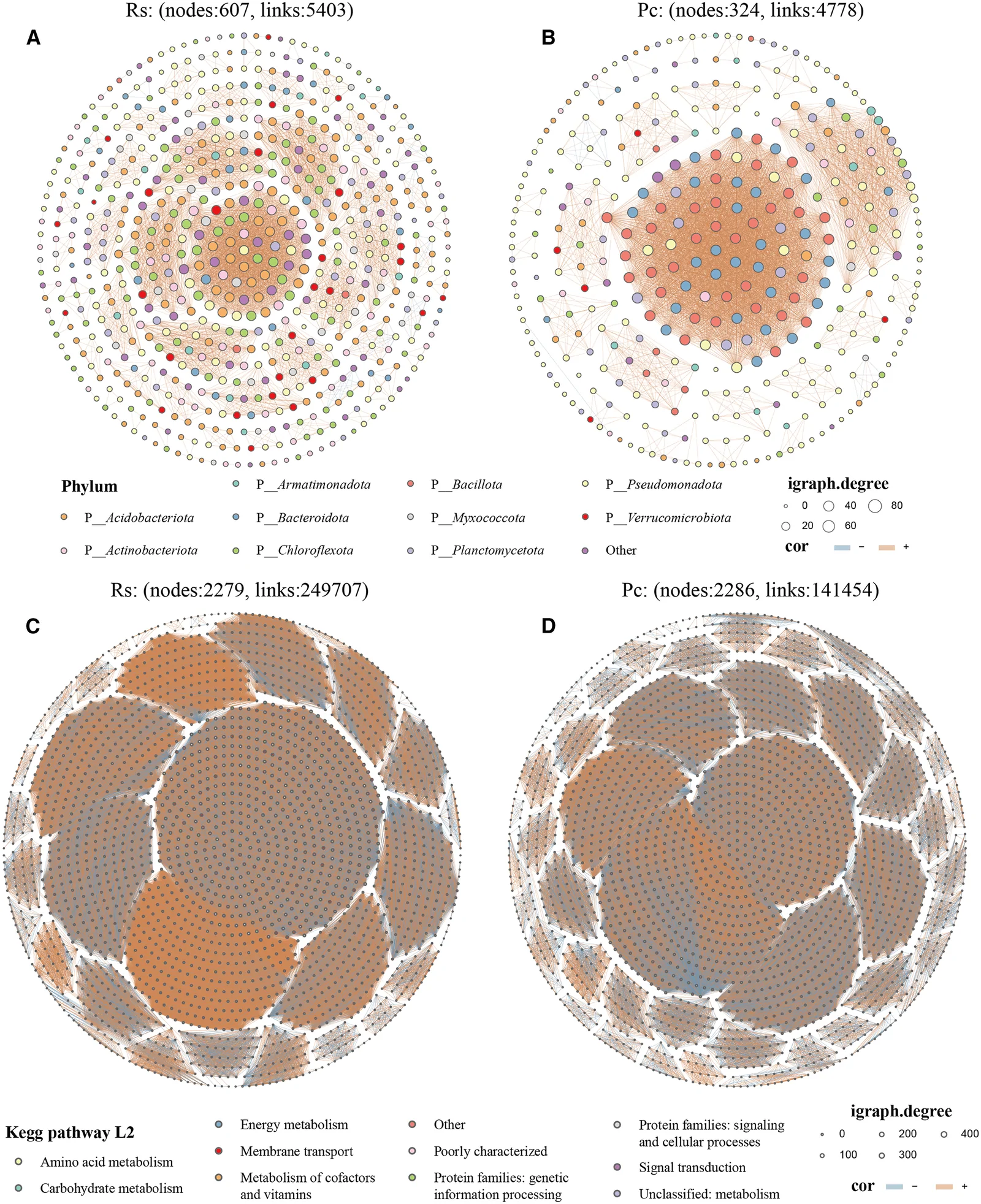
Niche-specific co-occurrence network analysis Pc: *Pogonatum cirratum* endophytic prokaryotic communities, Rs: rhizosphere soil prokaryotic communities. (A) Species co-occurrence network in Rs, where nodes represent ASVs and edges indicate significant correlations. Node size/color reflects taxonomic affiliation (phylum) and connectivity (igraph.degree). (B) Species co-occurrence network in Pc, with node properties as in (A). (C) Functional co-occurrence network in Rs, where nodes represent KO numbers and edges indicate significant correlations. Node size/color reflects KEGG pathway level 2 and connectivity (igraph.degree). (D) Functional co-occurrence network in Pc, with node properties as in (C).

To dissect niche-specific microbial interactions, we constructed niche-specific co-occurrence networks for prokaryotic species and functions separately in the Rs and Pc of *P. cirratum*. Amplicon sequence variants (ASVs) and KO numbers were filtered by average abundance (<0.01%) and sample presence (<3 ASVs per five samples within each niche) to ensure ecological relevance. In the Rs species co-occurrence network (Figure 3A), there were 607 nodes (ASVs) and 5,403 edges, with 5,320 positive edges and 83 negative edges. Key topological features included connectome (edge density) of 0.0294, average degree of 17.80, and modularity index of 0.852. Positive correlations dominated (98.46% of all relationships), with negative correlations accounting for only 1.54%. This high taxonomic diversity yet low connectome and average degree align with the rhizosphere’s heterogeneous nutrient landscape, where microbial taxa engage in loosely structured, nutrient-driven interactions to exploit diverse carbon and nitrogen sources. The high modularity (0.852) further suggests niche partitioning among Rs microbes in response to diverse soil resources. In Pc species co-occurrence network (Figure 3B), there were 324 nodes (ASVs) and 4,778 edges. Topological characteristics included a modularity index of 0.467, an average degree of 29.49, and a network density of 0.0913. Positive correlations were highly prevalent (99.39%), with negative correlations at 0.61%. *Pseudomonadota* was the predominant phylum, reflecting its central role in Pc microbial assemblages. Despite lower taxonomic richness, the high connectance and average degree, coupled with near-exclusive positive correlations, reflect strong host-mediated filtering within moss tissues, selecting for a specialized subset of microbes capable of intimate, cooperative interactions to support host fitness. The low modularity (0.467) indicates a more integrated community, driven by uniform selective pressure within the host tissue environment. Both Rs and Pc species networks were dominated by positive correlations, indicating prevalent cooperative interactions within each niche. Rs harbored a more diverse taxonomic composition (607 vs. 324 nodes), while Pc exhibited higher modularity, suggesting stronger niche filtering of microbial taxa in the endophytic environment.^50^^,^^51^^,^^52^

In Rs prokaryotic functional co-occurrence network (Figure 3C), there were 2,279 nodes (KOs) and 249,707 edges. Topological features included a modularity index of 0.607, an average degree of 219.14, and a density of 0.0962. Positive correlations accounted for 70.00%, while negative correlations accounted for 30.00%. The high connectivity (average degree and density) and broader coverage of metabolic pathways (e.g., energy metabolism and carbohydrate degradation) mirror the complex nutrient dynamics and microbial competition in rhizosphere soil. In the endophytic niche (Pc) prokaryotic functional network (Figure 3D), there were 2,286 nodes (KOs) and 141,454 edges. Topological characteristics included a modularity index of 0.795, an average degree of 123.76, and a density of 0.0542. Positive correlations made up 68.20%, with negative correlations at 31.80%. The higher modularity (0.795 vs. 0.607 in Rs) and enrichment of host-associated functions (e.g., signal transduction and xenobiotics degradation) highlight the endophytic niche’s reliance on microbe-host symbiosis, where prokaryotes have evolved specialized functions detoxify to xenobiotics compounds and mediate nutrient exchange with *P. cirratum*. The Rs functional network displayed higher connectivity (average degree and density), while the Pc functional network showed higher modularity, indicating niche-specific functional organization.

Collectively, these niche-specific co-occurrence networks demonstrate that *P. cirratum* prokaryotic communities develop distinct interaction patterns in response to their ecological contexts, with Rs favoring diverse, nutrient-driven interactions and Pc prioritizing host-adapted and specialized functions. These cooperative interactions likely support *P. cirratum*’s growth and environmental adaptation, underscoring the critical role of ecological context in shaping plant-microbe symbioses and moss-microbe interactions in terrestrial ecosystems.

### Metagenomic analysis reveals ecological functional differences in prokaryotic communities

To validate and deepen our understanding of prokaryotic functional differences beyond amplicon-based predictions, we performed KEGG pathway enrichment analysis^53^^,^^54^ on the metagenomic functional profiles. This analysis identified several shared enriched pathways in prokaryotic communities from the Rs and Pc of *P. cirratum*. These pathways encompassed biosynthesis of cofactors, carbon metabolism, biosynthesis of amino acids, glycine, serine, and threonine metabolism, glyoxylate and dicarboxylate metabolism, and the bacterial secretion system (Figure S4). Such pathways underscore the pivotal role of these prokaryotes in energy metabolism and small-molecule biosynthesis, which are vital for nutrient cycling and plant growth promotion.^55^^,^^56^ Distinct functional profiles emerged between the two niches: endophytic prokaryotic communities (Pc) were enriched in pathways linked to biofilm formation, flagellar assembly, and the caulobacter cell cycle. This suggests these microbes may require enhanced mobility and colonization capabilities within moss tissues, potentially aiding *P. cirratum* in mitigating biotic and abiotic stresses and bolstering its resilience under challenging conditions.^57^^,^^58^ In contrast, rhizosphere soil prokaryotic communities (Rs) showed enrichment in pathways associated with amino sugar and nucleotide sugar metabolism, butanoate metabolism, and DNA replication. The prominence of amino sugar and nucleotide sugar metabolism likely stems from moss root secretions that support these metabolic pathways. Additionally, elevated butanoate metabolism indicates the involvement of these prokaryotes in decomposing complex organic matter in the rhizosphere.^59^^,^^60^^,^^61^

**Figure 4:**
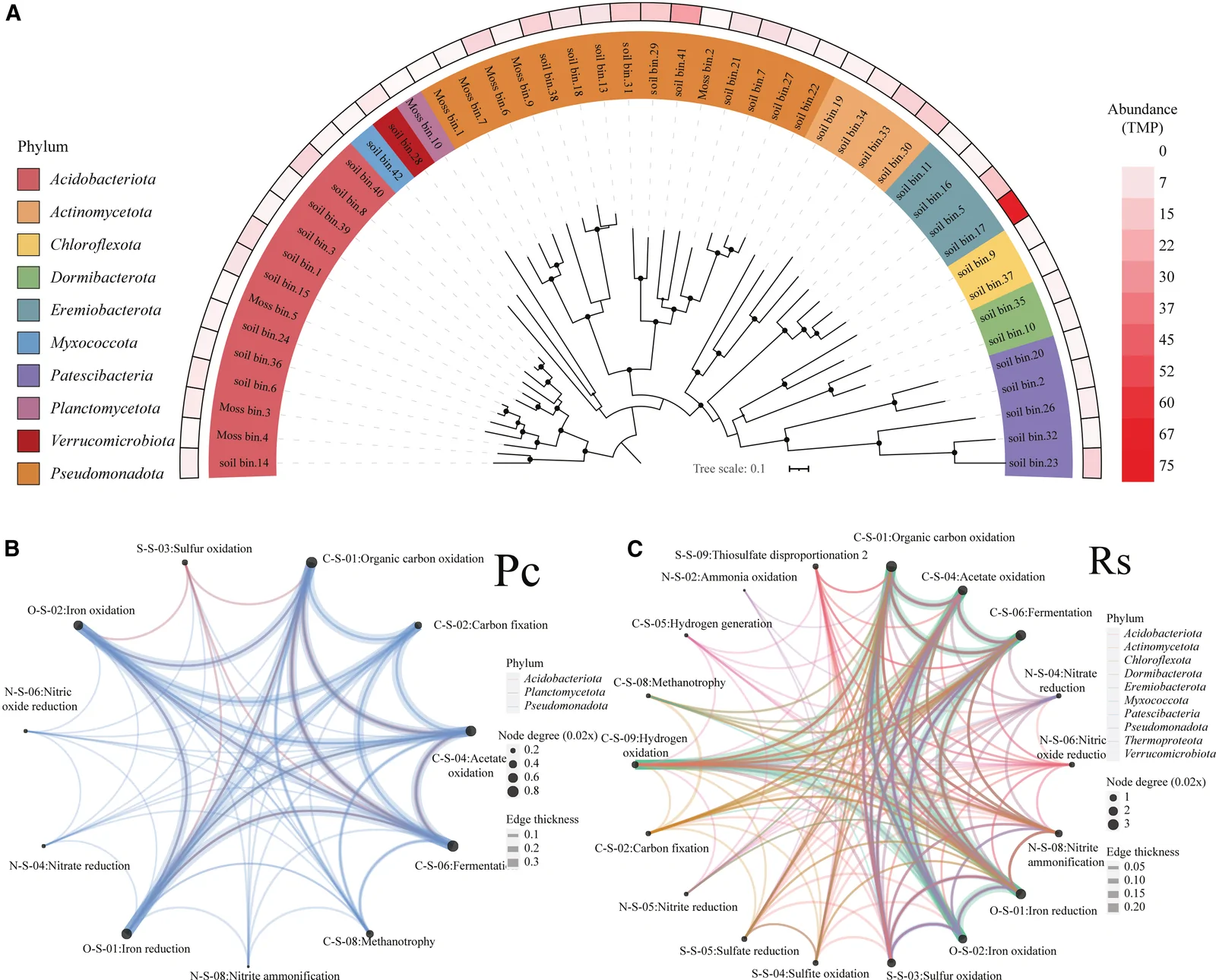
Ecological functional analysis of metagenomes Pc: *Pogonatum cirratum* endophytic prokaryotic communities, Rs: rhizosphere soil prokaryotic communities. (A) Phylogenetic tree of 48 bacterial MAGs, with bootstrap values greater than 80% indicated by black circles. (B) Functional network of the Pc prokaryotic community, where nodes represent individual steps in biogeochemical cycles, edges represent functional connections (enabled by organisms conducting both steps), node size reflects degree (number of connections), edge thickness reflects average gene coverage, and edge color reflects the taxonomy of the represented genome. (C) Functional network of the Rs prokaryotic community, with node/edge properties as in (B).

From the metagenomic data, 48 bacterial genomes (39 from Rs and 9 from Pc) and 1 archaeal genome (from Rs) were reconstructed (completeness ≥ 50% and contamination < 10%). These genomes belonged to 10 bacterial phyla and 1 archaeal phylum (Figure 4A and Table S1). Functional diversity analysis of these MAGs indicated significantly higher functional diversity in Pc samples compared to Rs samples (Figure S5). A functional network illustrating connections between ecological processes in the microbial community was constructed using METABOLIC software. In the endophytic niche (Pc), the phylum *Pseudomonadota* was the dominant group driving ecological functions, contributing 78.79% of the total functional weight (Figure 4B and Table S2). *Pseudomonadota* is widely distributed in natural environments and plays a crucial role in nitrogen and carbon cycling, as well as biofilm formation, essential processes for plant growth and development.^22^ In contrast, the dominant metabolic groups in the rhizosphere soil (Rs) were more diverse, including *Pseudomonadota* (31.51%), *Acidobacteriota* (23.12%), *Actinomycetota* (15.49%), and *Eremiobacterota* (13.64%) (Figure 4C and Table S3), indicating their multifaceted roles in the soil ecosystem. This diversity may be linked to the highly complex soil environment, as moss rhizosphere soil is generally acidic.^62^^,^^63^ *Acidobacteriota* is well-adapted to acidic conditions and contributes to organic matter decomposition and nutrient cycling in the soil. *Actinomycetota*, known for antibiotic production, may enhance plant defense against pathogens and improve stress resistance.^64^

Notably, genes associated with iron cycling were detected in both niches, accounting for 14.60% and 14.00% of total metabolic weights in Rs and Pc samples, respectively (Figures S6 and S7). Iron is essential for plant growth, and while prior research has focused on iron cycling in rhizosphere soil, its presence within moss tissues suggests that endophytic prokaryotes may contribute significantly to iron acquisition and supply.^56^^,^^64^^,^^65^^,^^66^ This finding underscores a potentially unique adaptation in *Pogonatum cirratum*, meriting further exploration.

### Viral diversity and functional roles in *Pogonatum cirratum* across ecological niches

**Figure 5:**
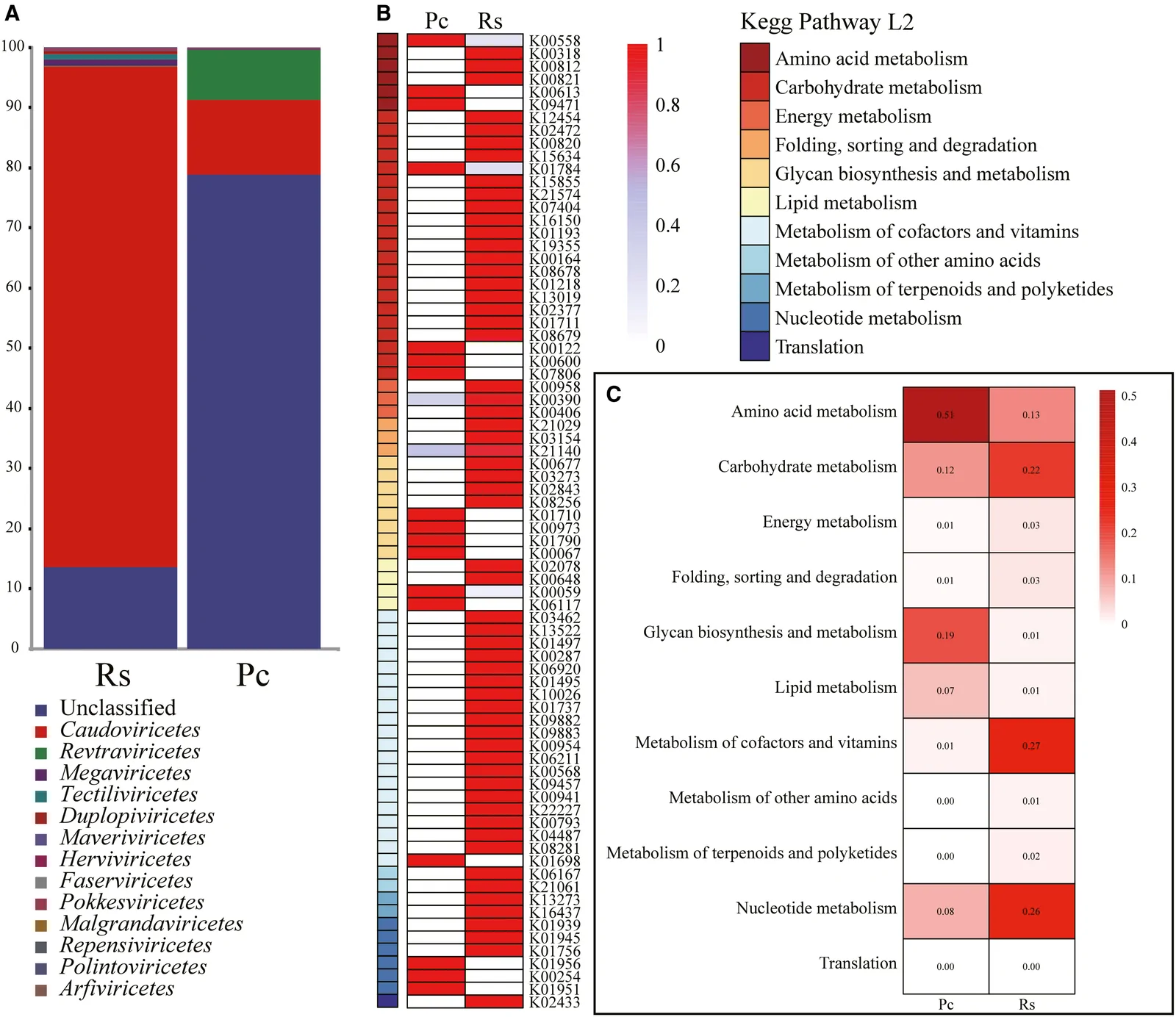
Viral composition and auxiliary metabolic gene analysis Pc: *Pogonatum cirratum* endophytic prokaryotic communities, Rs: rhizosphere soil prokaryotic communities. (A) Taxonomic composition of viral communities in Rs and Pc. (B) Relative abundance of auxiliary metabolic genes (AMGs) in Rs and Pc. (C) Relative abundance of AMGs mapped to KEGG pathway level 2 in Rs and Pc.

To investigate the largely understudied viral communities associated with *P. cirratum* and their potential auxiliary metabolic roles, we analyzed viral sequences recovered from the metagenomic data. Metagenomic analysis revealed a higher abundance of viral fragments in the rhizosphere soil (Rs, 2,096) than in *P. cirratum* moss tissues (Pc, 1,735). Taxonomically, Rs was dominated by *Caudoviricetes*, which is consistent with the widely reported finding that tailed phages prevail in various soil ecosystems including agricultural fields, grasslands, and forest soils.^36^ By contrast, the viral community in moss tissues contained high abundances of both *Caudoviricetes* and *Revtraviricetes* (rarely detected in rhizosphere soils), clearly indicating distinct viral taxonomic partitioning across the two niches (Figure 5A).

To further explore AMGs and virus-host associations, we screened viral sequences longer than 5,000 bp using ViWrap software. Rhizosphere soil (Rs) exhibited a more diverse repertoire of AMGs, predominantly associated with carbohydrate metabolism, metabolism of cofactors and vitamins, and nucleotide metabolism (Figures 5B and 5C), mirroring the core AMG profiles of Pseudomonas phages documented in global soil viromes.^36^ These metabolic pathways underpin energy production and small-molecule biosynthesis, suggesting that Rs viruses may bolster energy and nutrient supply for moss development by reprogramming host central metabolism to accelerate the degradation of plant residues and root exudates.^31^^,^^67^ Conversely, viruses in moss tissues (Pc) were enriched in AMGs linked to amino acid metabolism and glycan biosynthesis and metabolism (Figure 5C), in sharp contrast to soil viruses. These functions are critical for energy homeostasis and stress resilience, implying Pc viruses potentially support moss physiological homeostasis, representing a specialized adaptive strategy of plant endophytic viruses to assist their symbiotic hosts.^68^

**Figure 6:**
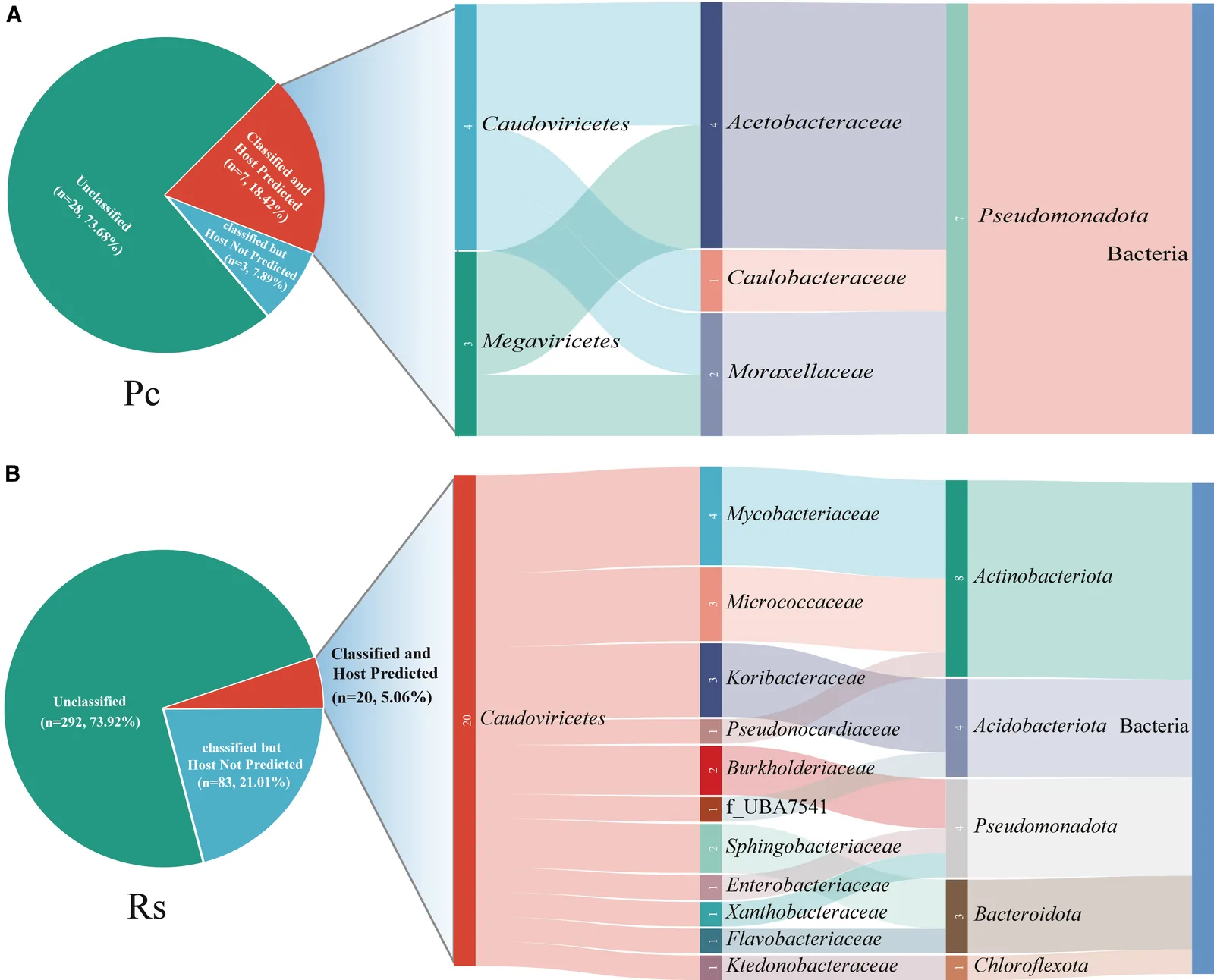
Virus-host predictions Pc: *Pogonatum cirratum* endophytic prokaryotic communities, Rs: rhizosphere soil prokaryotic communities. (A) Virus-host associations in Pc. (B) Virus-host associations in Rs.

Virus-host interactions, predicted via iPHoP software,^69^ showed that 18.42% of classified Pc viruses could be linked to host taxa, all of which were restricted to three families within *Pseudomonadota* (*Acetobacteraceae*, *Caulobacteraceae*, and *Moraxellaceae*), with no evidence of cross-phylum infection (Figure 6A). In contrast, 5.06% of classified Rs viruses had identifiable hosts, which spanned 11 families across 6 bacterial phyla (*Actinobacteriota*, *Acidobacteriota*, *Pseudomonadota*, *Bacteroidota*, and *Chloroflexota*) (Figure 6B). Collectively, this confirms that rhizosphere *Caudoviricetes* retain the typical broad host spectrum of soil phages,^36^ a trait that enhances their survival in highly diverse and dynamic soil microbial communities, while endophytic viruses have evolved extreme host specificity driven by long-term co-evolution in closed plant tissues.^70^ Notably, a large fraction of Pc viruses remained unclassified, highlighting the vast, unexplored viral diversity in non-vascular plants and their potential ecological roles, a phenomenon also widely reported in virome studies of other plants,^71^ underscoring the need to further investigate their functions and interactions with prokaryotic communities.^67^

### Niche-specific differences in secondary metabolite gene clusters of prokaryotes in *Pogonatum cirratum*

**Figure 7:**
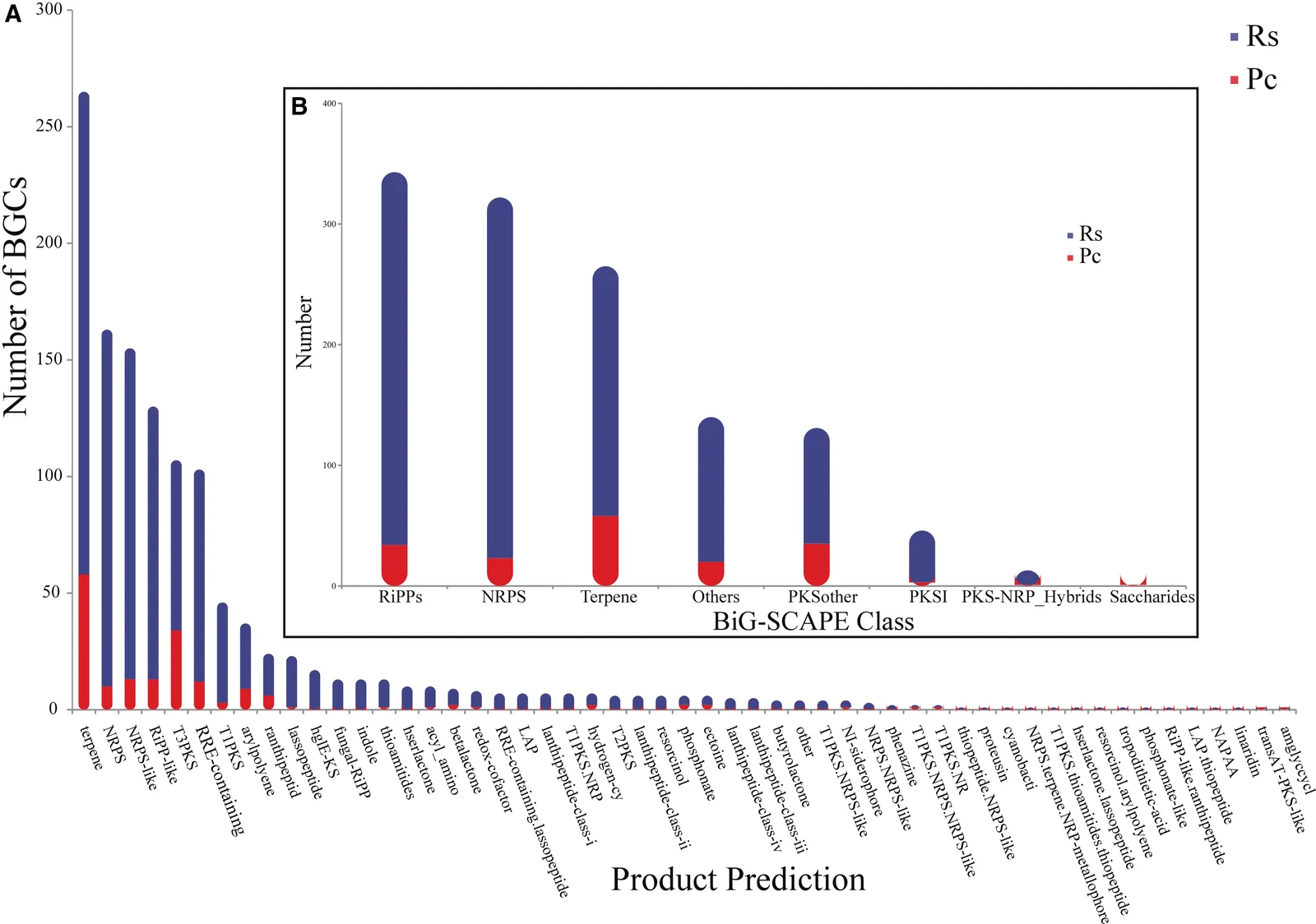
Analysis of prokaryotic secondary metabolite gene clusters Pc: *Pogonatum cirratum* endophytic prokaryotic communities, Rs: rhizosphere soil prokaryotic communities. (A) Distribution of biosynthesis gene clusters (BGCs) in Rs and Pc, where the *x* axis represents predicted metabolite classes and the *y* axis represents the number of BGCs. (B) BiG-SCAPE clustering analysis of BGCs, with the *x* axis indicating cluster classes and the *y* axis indicating the number of clusters in Rs (blue) and Pc (red).

To explore the secondary metabolite biosynthetic potential of prokaryotic communities in the two niches, we predicted and compared biosynthetic gene clusters (BGCs) from the metagenomic data. Metagenomic analysis revealed clear differences in the abundance and composition of BGCs between prokaryotic communities from the Rs and Pc of *P. cirratum*. A total of 1,086 BGCs were identified in the rhizosphere soil, whereas only 175 BGCs were detected in the endophytic community, which may indicate a far richer reservoir of secondary metabolite potential in the rhizosphere (Figure 7A). In Rs, the most abundant BGCs included terpene (19.06%), nonribosomal peptide synthetases (NRPS: 14.09%), NRPS-like (13.08%), and RiPP-like (10.77%). In contrast, within the endophytic prokaryotic communities, terpene (33.14%) and type III polyketide synthases (T3PKS: 19.43%) were dominant. This diversity likely stems from the combined influence of soil nutrients and moss root exudates, reflecting the rhizosphere’s role in supporting moss defense against pathogens and meeting growth demands.^72^

Biosynthetic Gene‑Similarity Clustering And Prediction Engine (BiG-SCAPE) clustering further differentiated niche-specific BGC profiles (Figure 7B). In Rs, clusters were primarily categorized as ribosomally synthesized and post-translationally modified peptides (RiPPs: 28.45%), NRPS (27.53%), and terpene (19.06%). In Pc, the major clusters included terpene (33.14%), RiPPs (19.43%), PKSother (20.00%), and NRPS (13.14%). RiPPs are the most abundant BGCs in both niches, exhibit diverse structures and biological activities, and have been proposed to play important roles in regulating plant secondary metabolism and enhancing defense against pathogens.^73^^,^^74^ Prediction of BGCs within MAGs indicated that *Acidobacteriota* and *Pseudomonadota* may encode a large number of BGCs (Table S2). This finding suggests that these prokaryotes may produce secondary metabolites that modulate *P. cirratum*’s physiological functions, thereby contribute to enabling the moss to adapt to its growth niche. For example, *Acidobacteriota*, adapted to acidic rhizosphere conditions, may contribute to organic matter decomposition and nutrient cycling via secondary metabolites, while *Pseudomonadota*, dominant in both niches, likely mediate plant-microbe interactions through metabolite secretion.

### Limitations of the study

Several limitations of this study should be acknowledged. Sampling was restricted to a single site (Danxia Mountain Nature Reserve) with only five biological replicates per niche, and metagenomic analyses were performed on a single pooled composite sample per niche rather than individual replicates. Additionally, fungal communities were not included in this study due to persistent technical challenges in amplifying high-quality fungal Internal Transcribed Spacer (ITS) sequences from moss tissue samples, which contained an extremely high proportion of host genomic DNA. Future studies incorporating multi-site sampling, replicate-level metagenomics, integrated fungal community characterization, and experimental validation will help address these gaps.

Despite the limitations of pooled metagenomic sampling, the consistency between our amplicon and metagenomic results provides strong support for the core findings of this study.

Mosses, as pivotal components of terrestrial ecosystems, rely on prokaryotes and viruses to sustain their growth, development, and ecological functions. This is the first study to employ amplicon and metagenomic sequencing technologies to systematically characterize the diversity and functional roles of prokaryotic and viral communities associated with *P. cirratum*, and to disentangle their potential regulatory impacts on the host moss.

This study reveals that deterministic processes (e.g., niche filtering and host-mediated selection) predominate in the assembly of prokaryotic communities in *P. cirratum*. Notably, while all co-occurrence networks are dominated by positive correlations, distinct niche-specific patterns emerge: in species co-occurrence networks, the proportion of positive correlations in endophytic microbial communities (Pc) is higher than that in rhizosphere soil microbial communities (Rs); in contrast, in functional co-occurrence networks, the proportion of positive correlations in rhizosphere soil (Rs) exceeds that in the endophytic niche (Pc). This divergent correlation pattern across network types indicates that the moss adjusts the intensity of microbial cooperation according to niche-specific functional demands. Furthermore, we observed pronounced niche-specific differences in the taxonomic composition and functional profiles of prokaryotic and viral communities between the rhizosphere soil and host moss tissues. These differences highlight their distinct contributions to the moss’s growth and environmental adaptability: despite lower taxonomic diversity, the endophytic prokaryotic community exhibits broader functional versatility, which likely enables the host to cope with environmental challenges. Of particular note, prokaryote-mediated iron cycling emerges as a conserved key process in both niches, though its underlying mechanisms remain to be further investigated. Additionally, secondary metabolites (e.g., RiPPs and terpenes) produced by prokaryotic communities appear vital for supporting moss growth and stress tolerance.

Collectively, this study enhances our understanding of the diversity, interactions, and ecological functions of prokaryotic and viral communities in moss systems. By elucidating the potential mechanisms driving plant-microbe interactions, it establishes a mechanistic framework for investigating plant-microbe/viral dynamics and highlights microbial roles in terrestrial ecosystem resilience.

## Resource availability

### Lead contact

Further information and requests for resources should be directed to and will be fulfilled by the lead contact, Wei-Qiu Liu (lsslwq@mail.sysu.edu.cn).

### Materials availability

This study did not generate new unique plasmids, microbial strains, moss germplasm, or custom reagents.

### Data and code availability

• Data: All raw 16S rRNA amplicon and metagenomic sequencing reads generated in this work have been deposited in NCBI BioProject under accession number PRJNA1189183 (https://www.ncbi.nlm.nih.gov/bioproject/PRJNA1189183/). This accession number is listed in the key resources table.
• Code: No custom public code repository was created for this study; all open-source software versions are fully documented in the key resources table.

## Acknowledgments

This study was supported by funding from the 10.13039/501100001809National Natural Science Foundation of China (nos. 32271565 and 31370490) and the Special Foundation for National Park Construction (2021GJGY034). We extend our sincere appreciation to the staff at Danxia Mountain Nature Reserve for their invaluable assistance and gracious permission granted for sample collection. Computation was performed on the Secevo HPC cluster of the School of Ecology, Sun Yat-Sen University.

## Author contributions

Conceptualization, C.-J.H., W.-Q.L., and W.-J.L.; writing – original draft and editing, C.-J.H. and M.A.; software and formal analysis, C.-J.H., X.-Q.L., W.-H.L., and M.-X.L.; investigation, J.-H.L., C.-G.X., Y.H., S.C., and J.-Y.C.; supervision, validation, and funding acquisition, W.-J.L. and W.-Q.L. All authors read and approved the final manuscript.

## Declaration of interests

The authors declare that they have no known competing financial interests or personal relationships that could have appeared to influence the work reported in this paper.

## STAR★Methods

### Key resources table

**Table:** 

REAGENT or RESOURCE	SOURCE	IDENTIFIER
**Biological samples**		

moss tissue and Rhizosphere soil of *P. cirratum* (Rs)	Danxia Mountain Nature Reserve, Guangdong, China	longitude 113°36′25″-113°47′53″E, latitude 24°51′48″-25°04′12″N

**Deposited data**		

16S rRNA amplicon sequencing raw reads and Metagenomic sequencing raw reads	BioProject accession number is PRJNA1189183	https://www.ncbi.nlm.nih.gov/bioproject/PRJNA1189183/

**Software and algorithms**		

QIIME2 v2023.2	N/A	https://qiime2.org/
DADA2	N/A	https://benjjneb.github.io/dada2/
SILVA database v138	N/A	https://www.arb-silva.de/
fastp v0.12.4	N/A	https://github.com/OpenGene/fastp
MetaWRAP v1.3	N/A	https://github.com/bxlab/metaWRAP
CheckM v1.2.0	N/A	https://github.com/Ecogenomics/CheckM
GTDB-Tk v2.3.0 (GTDB r214)	N/A	https://github.com/Ecogenomics/GTDBTk
geNomad v1.7.0	N/A	https://github.com/apcamargo/genomad
ViWrap v1.3.0	N/A	https://github.com/AnantharamanLab/ViWrap
Prodigal v2.6.3	N/A	https://github.com/hyattpd/Prodigal
Kofamscan v1.3.0	N/A	https://github.com/takaram/kofam_scan
METABOLIC v4.0	N/A	https://github.com/AnantharamanLab/METABOLIC
antiSMASH v7.0.0	N/A	https://antismash.secondarymetabolites.org/
BiG-SCAPE v1.1.9	N/A	https://github.com/medema-group/BiG-SCAPE
IQ-Tree v2.0.3	N/A	http://www.iqtree.org/
iTOL	N/A	https://itol.embl.de/
PICRUSt2	N/A	https://github.com/picrust/picrust2
R vegan	N/A	https://cran.r-project.org/package=vegan
R ggClusterNet2	N/A	https://github.com/taowenmicro/ggClusterNet
R clusterProfiler	N/A	https://bioconductor.org/packages/clusterProfiler
R FEAST	N/A	https://github.com/cozygene/FEAST
RStudio v4.3.3	N/A	https://www.rstudio.com/
LC-Bio Cloud Platform	N/A	https://www.omicstudio.cn/tool?order=complex
Majorbio Cloud Platform	N/A	https://cloud.majorbio.com/
Wekemo Bioincloud Platform	N/A	https://www.bioincloud.tech/#/
Hiplot Cloud Platform	N/A	https://hiplot.com.cn/

### Method details

#### Materials and methods

##### Sample collection

Samples were collected in July 2023 from the Danxia Mountain Nature Reserve in Guangdong, China (longitude 113°36′25″-113°47′53″E, latitude 24°51′48″-25°04′12″N). A systematic sampling transect was pre-established across the entire study area, and all sampling sites were selected as well-established, large monospecific stands of *Pogonatum cirratum* to eliminate confounding effects from co-occurring bryophyte species. Adjacent sampling sites were separated by a minimum distance of 200 m to minimize spatial autocorrelation. Well-grown *P. cirratum* was selected from five different roadside forest slopes. Moss tissues were carefully harvested using sterilized tweezers and washed three times with sterile water. Cleaned moss samples were then placed into sterile sampling bags. Following the collection of moss tissues, surface litter was gently removed. Shallow rhizosphere soil (0-3 cm depth) was excavated using a sterile sampling spoon and transferred into separate sterile sampling bags. In total, five *P. cirratum* tissue samples (denoted as Pc) and five rhizosphere soil samples (denoted as Rs) were collected. All samples were immediately stored in a cooler at 4°C for transport to the laboratory and then preserved in a -80°C freezer to ensure their integrity for subsequent analysis.

#### DNA extraction and 16S rRNA gene amplicon sequencing analysis

Samples were transported on dry ice to Sangon Biotech Co., Ltd. (Shanghai, China) for total DNA extraction. The V4-V5 region of the 16S rRNA gene was amplified using universal primers 515F (5′-GTGCCAGCMGCCGCGGTAA-3′) and 909R (5′-CCCCGYCAATTCMTTTRAGT-3′).^75^ The amplified products were sequenced on the MiSeq platform (Illumina). Raw reads were demultiplexed and quality-filtered using QIIME2 v2023.2^76^ with default parameters (Phred quality score ≥20, read length ≥200 bp); Chimeric sequences were identified and removed using DADA2; taxonomic classification was performed using the SILVA v138^77^ databases with a 99% identity cut-off. Downstream analyses, including microbial community diversity alpha/beta assessment (R vegan^78^), functional prediction (PICRUSt2^79^), co-occurrence network construction (R ggClusterNet2^80^), community assembly mechanisms,^81^ enrichment analysis (R clusterProfiler^82^), source tracking analysis (R FEAST^83^), were performed using RStudio 4.3.3 following the methods outlined by Liu et al.^84^ Data visualization was performed using the LC-Bio Cloud Platform,^85^ Majorbio Cloud Platform,^86^ Wekemo Bioincloud Platfrom,^87^ and Hiplot Cloud Platform (https://hiplot.com.cn/).

#### Metagenomic sequencing analysis

To comprehensively characterize the functional potential and viral diversity of prokaryotic communities in each niche, DNA from the five *P. cirratum* tissue samples and five rhizosphere soil samples was pooled separately to create one composite sample per niche. These composite samples were subsequently subjected to metagenomic sequencing on the MGI platform (DNBSEQ-T7). Quality control and data filtering of the raw data were performed using fastp software (version 0.12.4).^88^ Clean data were assembled into contigs, and genome bins were reconstructed using the standard workflow in MetaWRAP software (version 1.3).^89^ Medium-to-high-quality (≥50% completeness and <10% contamination) Metagenome-Assembled Genomes (MAGs) were selected using CheckM software (version 1.2.0).^90^ Classification and identification of MAGs were conducted using GTDB-Tk software (version 2.3.0) with the GTDB released 214 database.^91^ Viral sequences were identified from contigs using geNomad software (version 1.7.0), and ViWrap software (version 1.3.0) (parameters: --identify_method vb-vs, --input_length_limit 5000) was employed to screen viral sequences longer than 5000 bp for functional annotation and host prediction.^92^^,^^93^ Protein-coding genes were predicted using Prodigal software (version 2.6.3)^94^ with default parameters, followed by functional annotation using Kofamscan software (version 1.3.0).^95^ Prokaryotic metabolic networks of different ecological niches were constructed using METABOLIC software (version 4.0).^96^ Secondary metabolite biosynthetic gene clusters (BGCs) were predicted using antiSMASH software (version 7.0.0), and clustered using BiG-SCAPE software (version 1.1.9).^97^^,^^98^ Marker protein sequences from MAGs were extracted and aligned using GTDB-Tk software, and a maximum likelihood phylogenetic tree was constructed using IQ-Tree software (v 2.0.3)^99^ with parameters (-alrt 1000 -bb 1000 -nt AUTO). ModelFinder determined the best-fit model with parameters (-m MFP). The resulting phylogenetic tree was visualized and refined using the iTOL online software.^100^

### Quantification and statistical analysis

All statistical analyses were performed in R v4.3.3 using vegan, clusterProfiler, ggClusterNet2 and FEAST packages.

#### Alpha diversity comparison

Metrics: Observed species, ACE, Chao1, Shannon, Simpson, KEGG ortholog alpha diversity (functional diversity).

Test: Wilcoxon rank-sum test (non-parametric two-group comparison).

*n* = 5 biological replicates per niche (Rs rhizosphere soil, Pc moss endophyte).

Central measure: Median; dispersion: interquartile range (IQR). Significance threshold *p* < 0.01. Results presented in Figures 1B, 2B, S1, and S5.

#### Beta diversity

Distance metric: Bray-Curtis dissimilarity. Ordination via Principal Component Analysis (PCA).

Global group comparison: Wilcoxon rank-sum test, *n* = 5 per niche, *p* = 0.009 (prokaryotic taxa, Figure 1C); *p* = 0.01 (functional KO profiles, Figure 2C). Ellipses represent 95% confidence intervals.

#### Phylum relative abundance difference test

Two-group t-test for top 10 dominant phyla; significance marked ∗*p* < 0.05 (Figure 1A). *n* = 5 replicates per niche. Central tendency = mean relative abundance; dispersion = standard error of the mean (SEM).

#### LEfSe differential taxa identification

Linear discriminant analysis effect size, cutoff LDA score >5.0, Wilcoxon rank-sum test *p* < 0.05 (Figure 1D). *n* = 5 per niche.

#### Microbial source tracking (FEAST)

Quantified proportion of Pc endophytic taxa originating from Rs rhizosphere pool; output reported as percentage mean value (10.57%). Visualized in Figure S2.

#### Community assembly null model analysis (βNTI + RCbray)

NTI index for within-niche phylogenetic clustering; βNTI and RCbray to partition deterministic/homogeneous selection vs stochastic processes (dispersal limitation, homogenizing dispersal, undominated). Mean βNTI values reported for each niche (Rs = -3.35; Pc = -2.59). All pairwise comparisons between replicate samples (*n* = 5 per niche). Summarized in Figure S3.

#### KEGG level 2 functional pathway differential abundance (STAMP)

Welch’s t-test, 95% confidence intervals, *p* < 0.05 to filter significantly enriched pathways (Figure 2D). Central tendency = mean proportion; dispersion = 95% CI. *n* = 5 amplicon samples for PICRUSt2 prediction, pooled metagenomic data validated the pattern.

#### Co-occurrence network construction and topology statistics

ASV/KO abundance filtered by threshold (average abundance <0.01%, present in ≥3 of 5 samples). Network metrics: node count, edge count, edge density, average degree, modularity, positive/negative correlation ratio. All correlation tests are embedded within ggClusterNet2 pipeline. Visualized in Figures 3A–3D.

#### Metagenomic functional enrichment and MAG functional weight statistics

Functional contribution weight of each phylum to total biogeochemical pathways calculated via METABOLIC; iron cycling metabolic weight percentage reported as mean proportion across pooled metagenomic assemblies (Rs = 14.60%, Pc = 14.00%). No formal two-group significance test applied to pooled metagenomic composite samples (only one pooled metagenome per niche, n_pool = 1). MAG phylogeny bootstrap support ≥80% marked in Figure 4A.

#### Viral AMG, BGC and virus-host quantification

Count data of viral contigs, AMG relative abundance, BGC numbers were raw tally from metagenomic prediction pipelines (geNomad, ViWrap, antiSMASH, BiG-SCAPE). Virus-host matching ratio calculated as percentage of classified viral sequences linked to bacterial hosts (Figures 5, 6, and 7).

#### General statistical reporting rules

n always denotes number of independent biological replicate moss/rhizosphere soil samples (*n* = 5 for each niche).

Central tendency metrics: mean for relative abundance/functional proportion; median for alpha diversity indices.

Dispersion/precision measures: SEM, 95% confidence intervals, IQR, βNTI mean values as reported in text and figure captions.

All exact *p* values, test types, sample sizes are fully stated in main text results and figure legends as required.
