# Supplementary material for: Niche-driven divergence of prokaryotic and viral communities in Pogonatum cirratum
Source: iScience. 2026 Aug 13;29(9):117172. doi: 10.1016/j.isci.2026.117172 (PMC13495085; doi:10.1016/j.isci.2026.117172)
Supplement: Supplementary file 1 [file mmc1.pdf]

iScience, Volume *29*

Supplemental information

Niche-driven divergence of prokaryotic and viral communities in *Pogonatum cirratum*

Chao-Jian Hu, Muhammad Asif, Jin-Hui Liu, Cheng-Guang Xing, Xiao-Qing Luo, Wen-Hui Lian, Mei-Xiang Li, Yong Huo, Shu Cao, Jian-Yuan Chen, Wen-Jun Li, and Wei-Qiu Liu

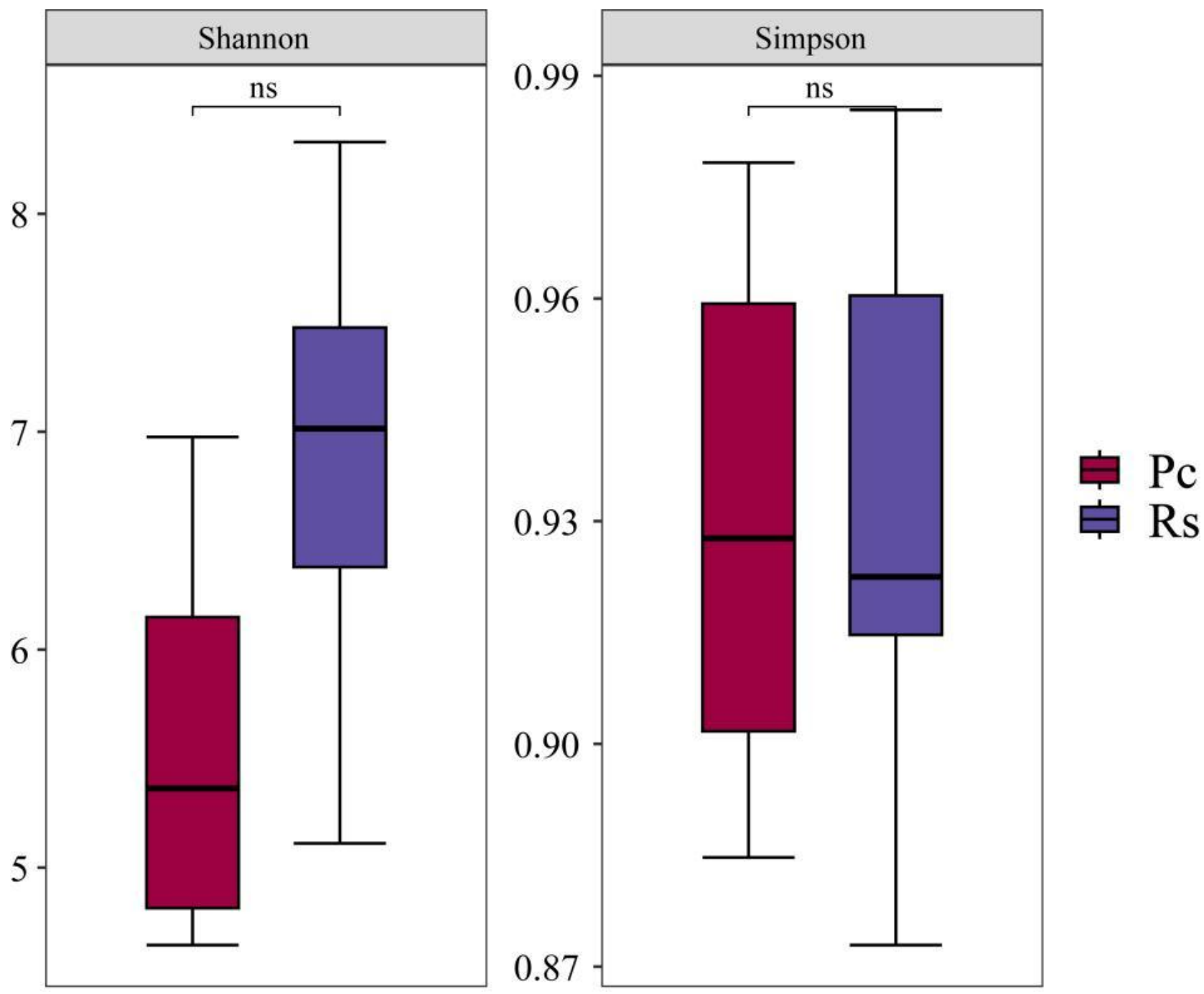

Figure S1. Comparison of the Shannon index and Simpson index between rhizosphere soil and endophytic prokaryotic communities. (Pc: *Pogonatum cirratum* endophytic prokaryotic communities, Rs: Rhizosphere soil prokaryotic communities; "ns" indicates no significant difference between groups)

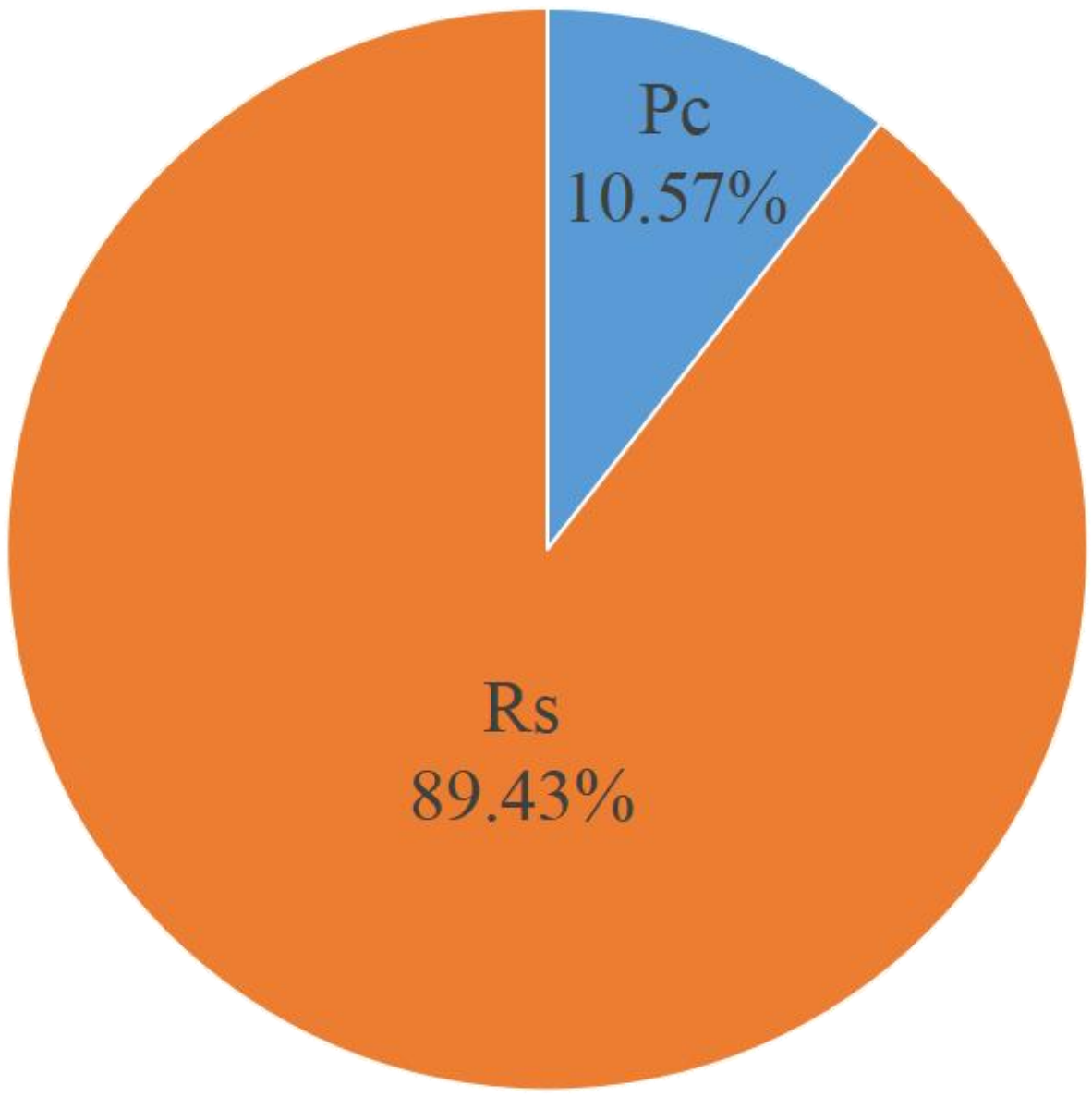

Figure S2. Source tracking of endophytic prokaryotic communities via FEAST software. FEAST prediction results showed that approximately 10.57% of microbes in the endophytic prokaryotic community (Pc) are derived from the rhizosphere soil prokaryotic community (Rs). (Pc: *Pogonatum cirratum* endophytic prokaryotic communities, Rs: Rhizosphere soil prokaryotic communities)

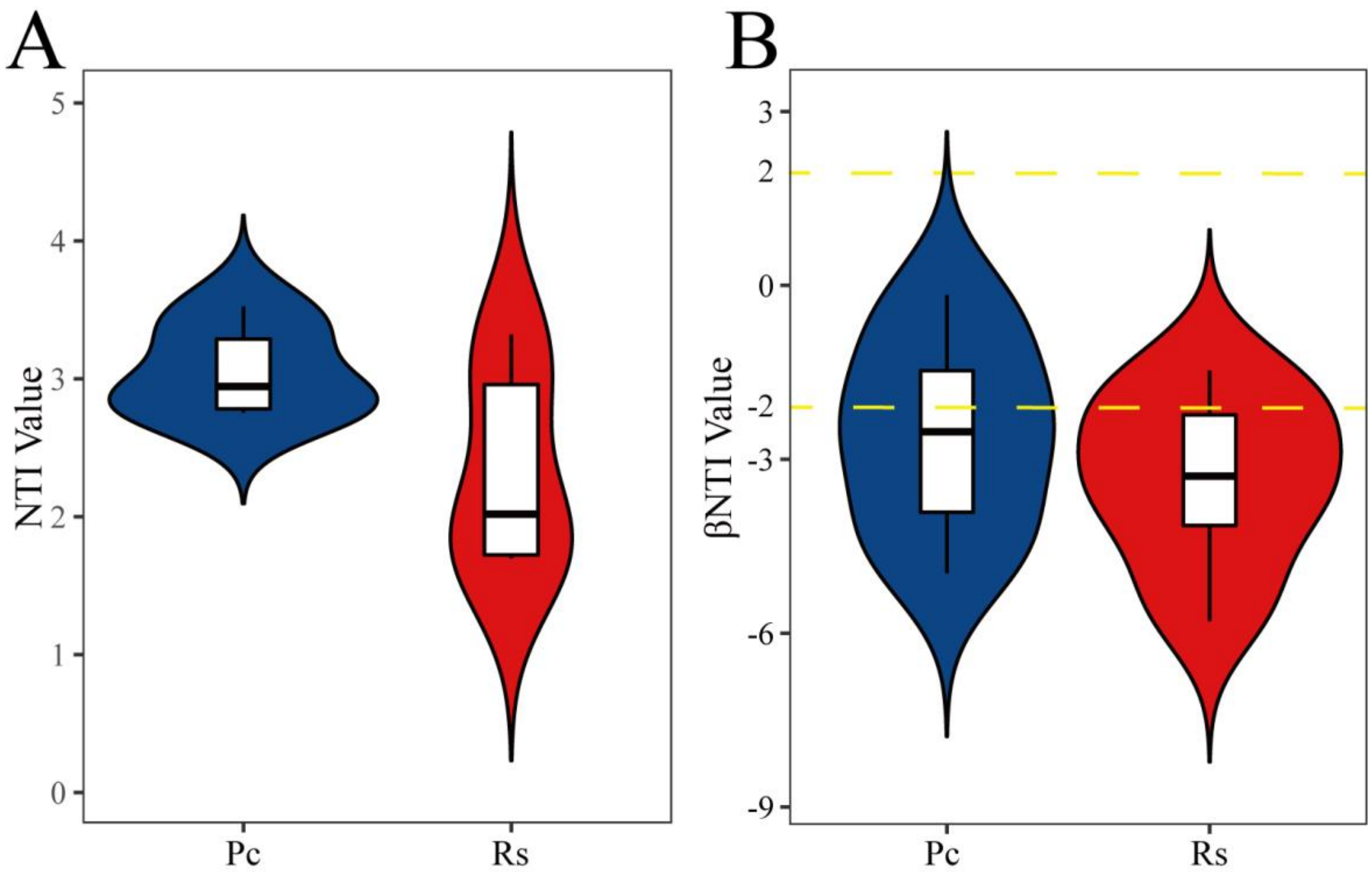

Figure S3. Analysis of community assembly mechanisms. A: Nearest Taxon Index (NTI) values of rhizosphere soil (Rs) and endophytic prokaryotic communities (Pc). B: Beta Normalized Taxonomic Index (βNTI) values of rhizosphere soil (Rs) and endophytic prokaryotic communities (Pc). (Pc: *Pogonatum cirratum* endophytic prokaryotic communities, Rs: Rhizosphere soil prokaryotic communities)

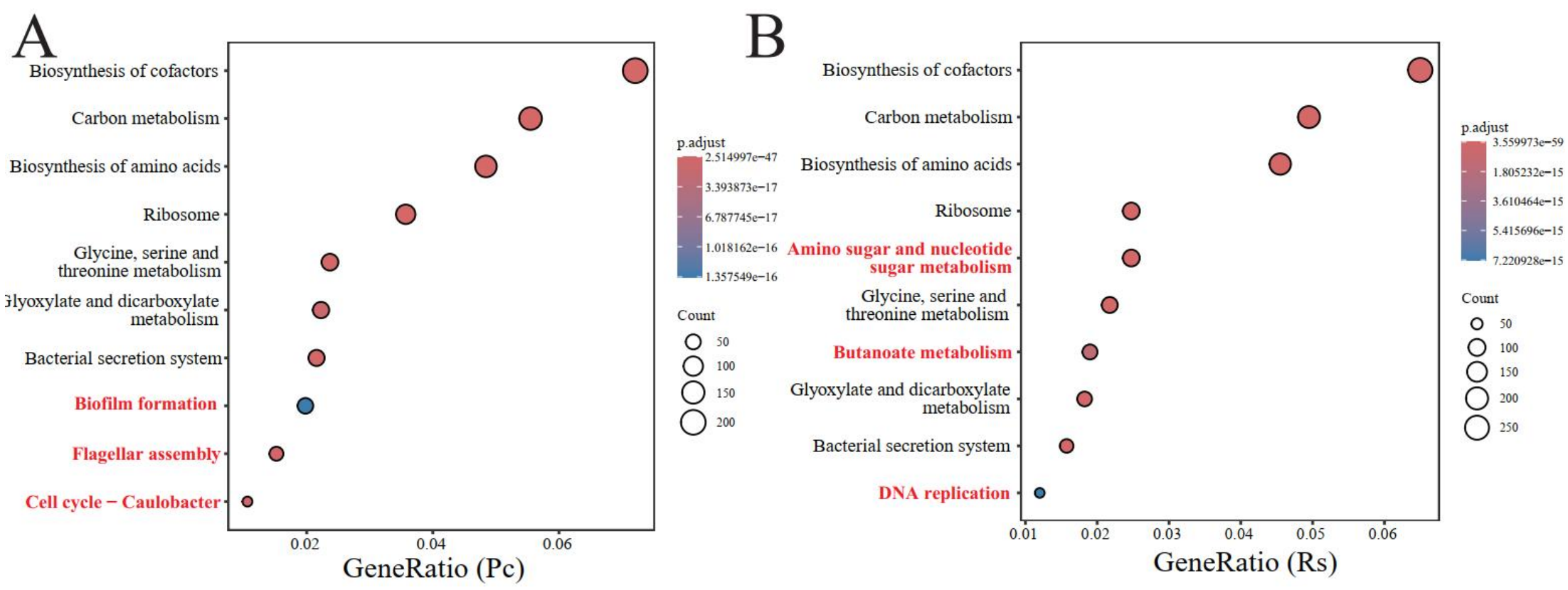

Figure S4. KEGG pathway enrichment analysis. A: Major enriched KEGG pathways in the endophytic prokaryotic community (Pc); B: TMajor enriched KEGG pathways in the rhizosphere soil prokaryotic community (Rs). (Pc: *Pogonatum cirratum* endophytic prokaryotic communities, Rs: Rhizosphere soil prokaryotic communities)

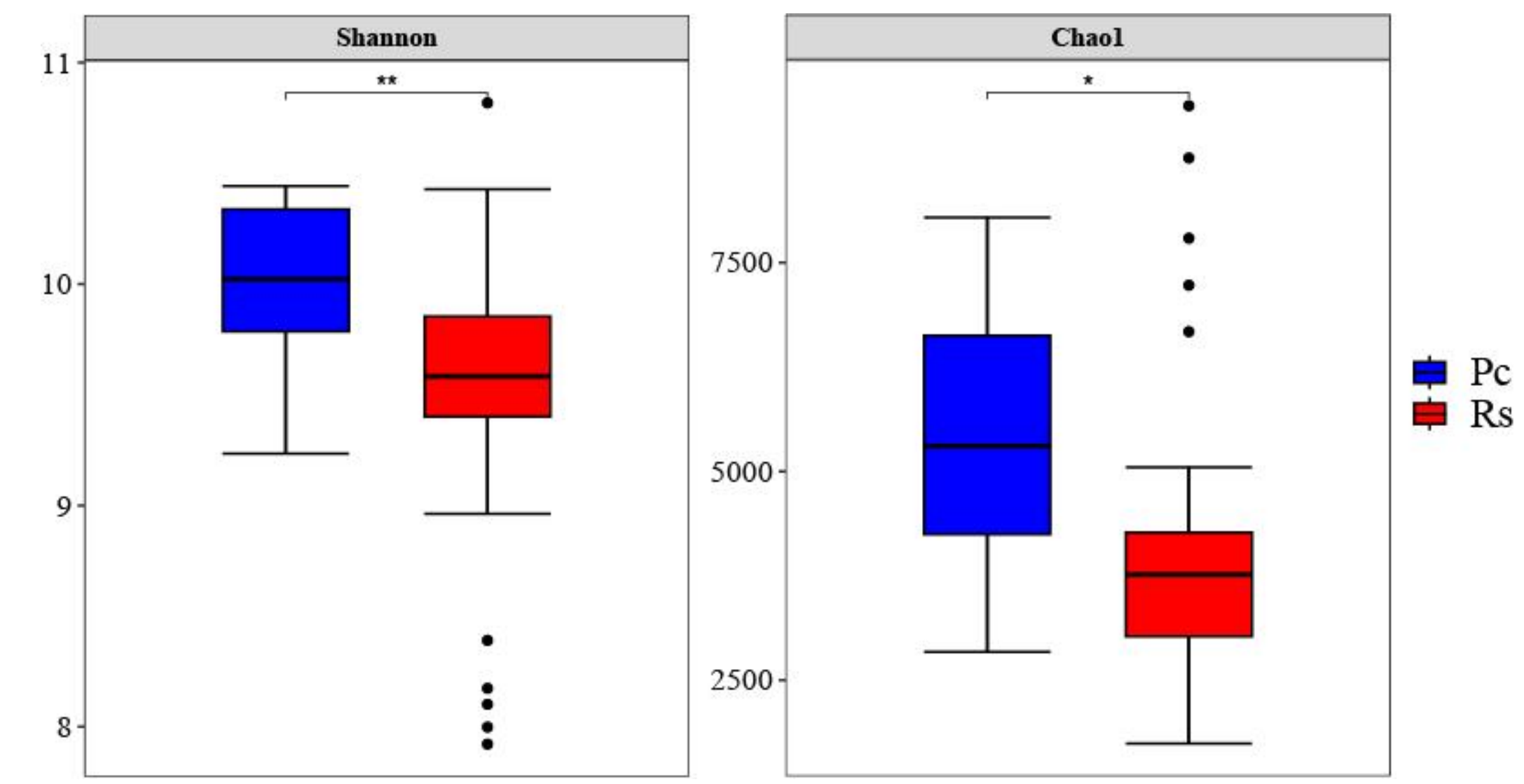

Figure S5 Functional alpha diversity analysis of Metagenome-Assembled Genomes (MAGs). (Statistical test: Wilcoxon rank-sum test, $p < 0.01$). (Pc: *Pogonatum cirratum* endophytic prokaryotic communities, Rs: Rhizosphere soil prokaryotic communities)

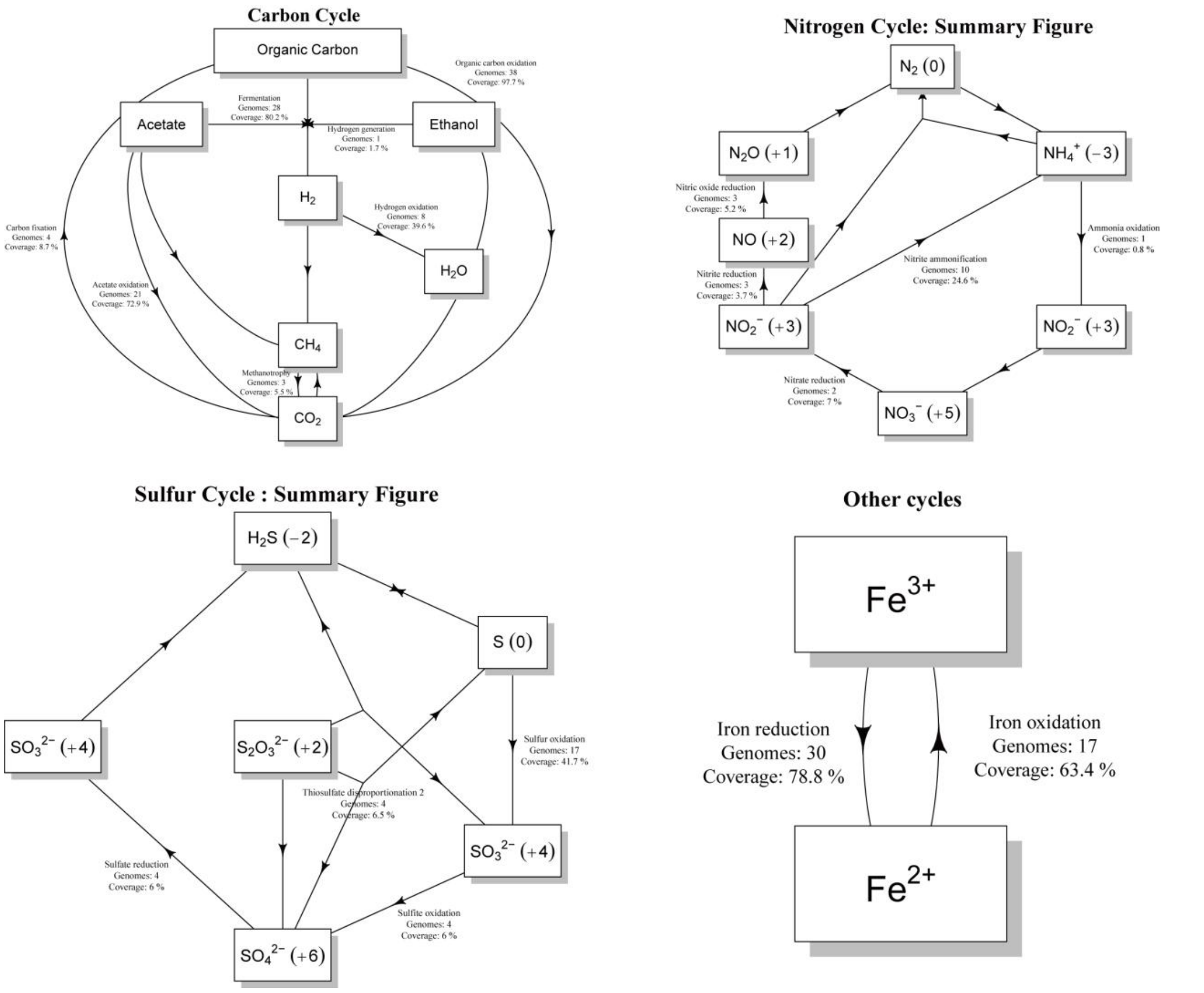

Figure S6. Potential ecological role of rhizosphere soil prokaryotic communities in biogeochemical processes. The number of MAGs containing each metabolic pathway and their coverage (%) was provided for each carbon, nitrogen, sulfur, and other cycles.

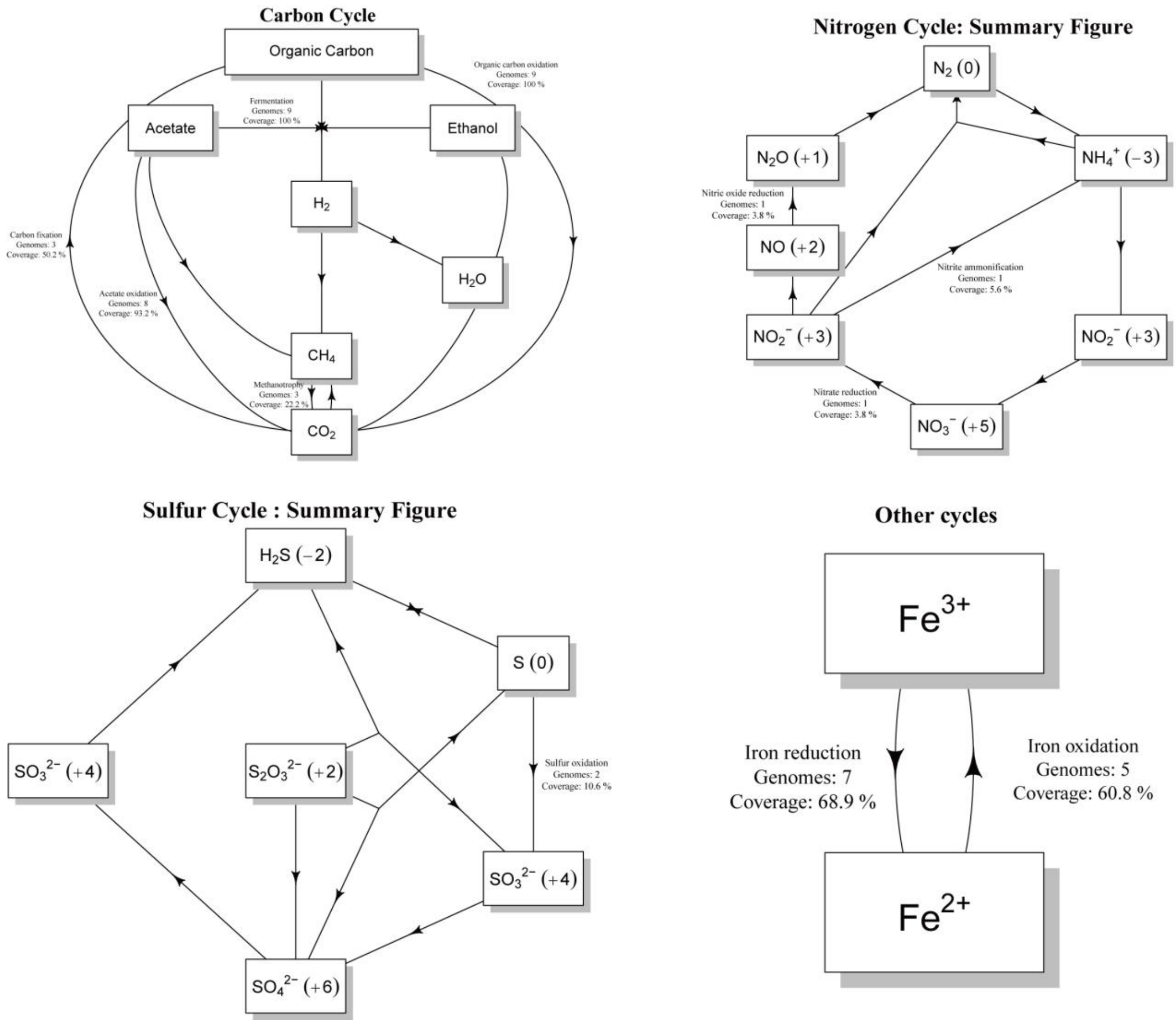

Figure S7. Potential ecological role of endophytic prokaryotic communities in biogeochemical processes. The number of MAGs containing each metabolic pathway and their coverage (%) was provided for each carbon, nitrogen, sulfur, and other cycles.
